# A systematic review of the use of theory in randomized controlled trials of audit and feedback

**DOI:** 10.1186/1748-5908-8-66

**Published:** 2013-06-10

**Authors:** Heather L Colquhoun, Jamie C Brehaut, Anne Sales, Noah Ivers, Jeremy Grimshaw, Susan Michie, Kelly Carroll, Mathieu Chalifoux, Kevin W Eva

**Affiliations:** 1 Ottawa Hospital Research Institute, Clinical Epidemiology Program, The Ottawa Hospital, General Campus, 501 Smyth Road, Centre for Practice Changing Research, Box 201B, Ottawa, Ontario, K1H 8L6, Canada; 2 Division of Nursing Business and Health Systems, Ann Arbor, MI, 48198, US; and VA Ann Arbor Healthcare System, Health Services Research and Development, University of Michigan School of Nursing, Ann Arbor, MI, 48105, USA; 3 Women’s College Hospital, Department of Family Medicine, Toronto, ON, M5S 1B2, Canada; 4 Department of Clinical, Educational and Health Psychology, University College London, London, WC1E 6BT, UK; 5 Centre for Health Education Scholarship, Department of Medicine, University of British Columbia, Vancouver, BC, V5Z 4E3, Canada

**Keywords:** Audit and feedback, Systematic review, Theory, Intervention design

## Abstract

**Background:**

Audit and feedback is one of the most widely used and promising interventions in implementation research, yet also one of the most variably effective. Understanding this variability has been limited in part by lack of attention to the theoretical and conceptual basis underlying audit and feedback. Examining the extent of theory use in studies of audit and feedback will yield better understanding of the causal pathways of audit and feedback effectiveness and inform efforts to optimize this important intervention.

**Methods:**

A total of 140 studies in the 2012 Cochrane update on audit and feedback interventions were independently reviewed by two investigators. Variables were extracted related to theory use in the study design, measurement, implementation or interpretation. Theory name, associated reference, and the location of theory use as reported in the study were extracted. Theories were organized by type (*e.g*., education, diffusion, organization, psychology), and theory utilization was classified into seven categories (justification, intervention design, pilot testing, evaluation, predictions, post hoc, other).

**Results:**

A total of 20 studies (14%) reported use of theory in any aspect of the study design, measurement, implementation or interpretation. In only 13 studies (9%) was a theory reportedly used to inform development of the intervention. A total of 18 different theories across educational, psychological, organizational and diffusion of innovation perspectives were identified. Rogers’ Diffusion of Innovations and Bandura’s Social Cognitive Theory were the most widely used (3.6% and 3%, respectively).

**Conclusions:**

The explicit use of theory in studies of audit and feedback was rare. A range of theories was found, but not consistency of theory use. Advancing our understanding of audit and feedback will require more attention to theoretically informed studies and intervention design.

## Background

The existence of a research to practice gap in healthcare is well established [1,2]. Audit and feedback is one of the most widely used interventions for decreasing this gap [3]. The term ‘Audit and feedback’ has been used generally to refer to a heterogeneous group of interventions that provide feedback on existing practice to healthcare providers. A&F can be defined as a summary of clinical performance (audit) over a specific period of time, and the provision of that summary (feedback) to individual practitioners, teams, or healthcare organizations [4].

The most recent 2012 Cochrane update for A&F interventions contained 140 randomized controlled trials of A&F [3]. Intervention effects ranged from substantially positive (70% increase in desired behavior) to negative (9% absolute decrease) with a median adjusted risk difference of 4.3% absolute increase and an interquartile range (IQR) of 0.5% to 16% [3]. This recent review was one of the first to attempt an examination of factors to explain the wide variation in A&F effectiveness. A meta-regression analysis found five A&F characteristics associated with effectiveness: source is a supervisor or colleague, is delivered more than once, is verbal and written, aims to decrease undesirable behavior as opposed to increase desirable behavior, and includes explicit targets and action plans. The authors, however, advise cautious interpretation of these conclusions due to the lack of direct comparisons and the lack of specificity in defining these characteristics. Ivers and colleagues conclude that unless we approach the study and reporting of A&F differently, we are unlikely to learn more from future Cochrane updates [3].

Our poor understanding of the variability in A&F may result from at least two important and related issues. First, A&F in the context of health provider behavior change is a heterogeneous group of activities resulting in complex interventions that are meant to function via multiple causal pathways. Second, we have a limited understanding of the theoretical mechanisms underlying any of these resulting causal pathways. A useful approach for defining and clarifying these potential mechanisms is the explicit use of theory. By providing summaries and predictions of causal pathways, the use of theory can help us understand these mechanisms and design more efficacious interventions [5,6]. Although the imperative for theory use in intervention development can be contested [7,8], there is considerable support for theory-based approaches [5,9-11]. However, several reviews have highlighted the current limited use of theory to guide implementation studies [3,12]. Theory-based syntheses of A&F interventions have been attempted in an effort to determine the relation between use of a specific theory and intervention effectiveness. To date, such efforts have been limited by insufficient reporting and use of theory [6]. To overcome this limitation and as a first step to understanding current practices for making explicit the causal mechanisms for A&F, we sought to explore more broadly which theories are being used in reporting A&F intervention studies.

We argue that the use of theory in study design, measurement, implementation or interpretation, as well as for intervention development, will lead to an improved understanding of the causal mechanisms by which interventions work, an improved framework to permit tailoring of interventions, and a better understanding of why some interventions fail while apparently similar approaches succeed [1,5]. Examining theory use for A&F studies is particularly important given its widespread use in implementation and the considerable variability in effects. This study aimed to determine the extent to which theory was explicitly reported in studies of A&F, the types of theories used, and the purpose to which the theory was put.

## Methods

We investigated the use of theory reported in randomized controlled trials of A&F. The dataset used for this review were all 140 randomized controlled trials included in the most recent Cochrane update of the effectiveness of audit and feedback [3]. For a full description of the Cochrane review methods, please see Ivers *et al*., 2012 [3]. Briefly, the search included the following databases from 1982 to 2011: Cochrane Central Register of Controlled Trials, MEDLINE, EMBASE, and CINAHL. The Science Citation Index and Social Sciences Citation Index were searched to identify studies citing studies included in the review. Randomized controlled trials that examined either health professional practice or patient outcomes were included. The trials had to consist of a summary of clinical performance over a specified period of time objectively measure health professional practice or patient outcomes, and A&F had to be considered a core aspect of the intervention for at least one intervention arm if a multifaceted intervention was used.

The variables collected for this review were as follows: the year the study was published, the name of theory as reported in the paper, the cited reference provided for the theory, the locations in the paper where the theory was introduced and subsequently discussed, and a classification of theory utilization using a seven-category rating scale developed based on expert opinion specifically for this study. In order to facilitate consistency, the original data extraction sheet and guide for all variables was piloted on five studies in the sample, resulting in refinements to various variable definitions. A second pilot of the revised form and guide was completed on 10 additional papers, resulting in a final form and guide. Extraction was shared among three individuals (HC, KC, MC) with two reviewers extracting for each study and one reviewer remaining consistent for all studies (HC). Each reviewer extracted separately, and disagreements were resolved through consensus.

In order to be classified as having used a theory, three conditions had to be met. First, the study had to state a theory by name, provide a reference for the named theory, and the theory had to adhere to our definition of a theory: ‘A set of concepts and/or statements with specification of how phenomena relate to each other. Theory provides an organizing description of a system that accounts for what is known, and explains and predicts phenomena’ [13]. Second, a theory reference had to pertain to the development of the theory, not a reference for an empirical study that had referenced or used the theory. Third, in order to be consistent with our definition of a theory, the theory had to include a set of concepts that described, explained and predicted phenomena including the relationships between the concepts. If, for example, an article stated that ‘learning theory’ was utilized in the study but did not name a specific theory or did not provide a theory reference, it was not counted as having utilized theory. If we were uncertain as to a named theory, we completed a search of the theory and used our definition of theory to guide our decisions about whether an actual theory had been used. The specific reference cited at the time the theory was first mentioned was used as the reference. To avoid underestimating theory use, where these three conditions were not met, we (HC, JB, KC) made a consensus decision as to whether a theory had actually been utilized or not.

Each theory used was placed in a descriptor category. The categories were determined by the prevailing description of the theory in the literature; *i.e*., Rogers’ Diffusion of Innovations is considered a diffusion theory. The categories were discussed by HC, JB, and KC to ensure consensus and were kept broad (*i.e*., ‘Psychology’) to avoid challenges in attempting to sub-categorize. The aim was to provide a general categorical summary.

Each study that utilized a theory was then placed into a category based on how the theory was used. This was achieved by reviewing how the study reported the use of the theory and considering each of the seven categories separately to determine which category/categories described the way in which the theory was used. These seven categories were derived by members of the study team (JB, KE). The categories and their descriptions were as follows: Justification: theory is discussed in the background/literature review/objectives section and is used to support study design/purpose; Intervention Design: the theory informed the intervention, either conceptually or by specifically influencing the design of the intervention; Pilot testing: theory was utilized within the study to guide pilot testing of the intervention; Evaluation: the theory or constructs outlined in the theory were used to guide outcomes measurement or develop the evaluation strategy; Predictions: at least one stated purpose of the study was to test the influence of a variable predicted to be relevant based on a given theory; Post hoc: theory was discussed in the discussion section for the purposes of supporting or explaining the results of the study; Other. The use of theory or constructs could be placed into any number of the categories that applied.

Inter-rater reliability was calculated for pre-consensus category assignment using the Kappa statistic to determine consistency across reviewers [14].

## Results

Table 1 provides a detailed list of all the theories identified along with their prevalence. Additional file 1: Table S1 outlines each individual study that used theory. Our systematic review found that 20 (14% of) studies explicitly reported using theory to some degree. A total of 18 different theories were present in the 20 studies that used theory. The maximum number of theories cited within a single study was three (n = 4); one study cited two theories, but most studies used only one theory (n = 15). The types of theories used fell into four broad fields of education, psychology, organization, or diffusion of innovation. The two most commonly used theories were Rogers’ Diffusion of Innovations [15] (n = 5) and Bandura’s Social Cognitive Theory [16] (n = 4); these were specifically used to justify aspects of the intervention in most cases (n = 3, n = 4 respectively).

**Table 1:** Theory names, theory authors, category, and degree of utilization

**Category**	**Theory name**	Number of times used*	Number of times used as part of intervention design**
**Theory author(s) and year**			
**Education**			
Knowles, 1984[17], 1990[18]	Adult Learning Theory	2	1
Coles & Holm, 1993[19]	Theory of Medical Education	1	1
**Diffusion**			
Rogers, 1983(3)[20], 1995(2)[15]	Diffusion of Innovations	5	3
Penland, 1997[21]	Technology Diffusion Theory	1	0
**Organization**			
Greenhalgh, Robert, Macfarlane, Bate, & Kyriakidou, 2004[22]	Diffusion of Innovations in Service Organizations	1	0
Lawler, 1976[23]	Lawler’s Organization Theory	1	1
Levitt, 1965[24]	Organization Development Theory	1	1
Kirkpatrick, 1967[25]	Kirkpatrick’s Hierarchy of Levels of Evaluation	1	0
**Psychology**			
Bandura, 1969[26], 1986(2)[16], 1997[27]	Social Cognitive Theory	4	4
Prochaska & DiClemente, 1982[28], 1986[29], 1992[30]	Transtheoretical Model	3	1
Fishbein, 1979[31], Fishbein & Ajzen, 1968[32]	Theory of Reasoned Action	2	1
Azjen, 1991^b^	Theory of Planned Behavior	1	1
Conner & Norman, 1996[33]	Social Cognitive Models	1	0
Green & Kreeuter, 1991[34]	Precede/Proceed Planning Model	1	1
Reason, 2000[35]	Human Error Theory	1	1
Ullman & Krasner, 1975[36]	Behavioral Psychology Theory	1	1
Perry, Baranowski, & Parcel, 1990[37]	Social Learning Theory	1	1
Social cognitive model: undefined^a^	Social Cognitive Models	1	0

^a^ Study indicated only that ‘social cognitive models’ were used but did not specify a reference.

^b^ Study used The Theory of Planned Behavior but did not provide a reference for the theory.

*These 29 uses of theory were located in 20 papers (14% of the 140 reviewed).

**These 18 uses of theory for intervention design were located in 13 papers (9% of the 140 reviewed).

All studies identified as having utilized theory met all three conditions for having used a theory with four exceptions [38-41]. One study named the theory used as The Theoretical Framework of Behavioral Psychology, but referenced Bandura’s Social Cognitive theory and used Social Cognitive Theory constructs in the study [38]. A second study named and utilized the Theory of Planned Behavior, but failed to provide an associated reference [39]. The two remaining studies stated that Social Cognitive Models were used [40,41], but did not state specifically which one and failed to provide a reference in one case [41]. In all four cases, the studies were classified as having used theory. These inclusion decisions were made based on consensus by HC, JB, and KC under the rationale that although they did not meet all three conditions, the study methods were clearly informed by the theories.

Table 2 provides a summary of the nine categories of theory use. All categories of theory use were represented, with the exception of ‘pilot testing’ and ‘other.’ The inter-rater reliability for the reviewers’ pre-consensus category assignment was calculated as Kappa = 0.93 (p<0.0001), 95% CI (0.88-0.98). A total of 6 of the 20 studies reporting use of theory used theory as a justification for the study [38,42-46], with one of these six studies only using theory as a justification for the study (*i.e*., in the introduction section) [45], never re-visiting the theory again. Eleven of the studies used theory as a post hoc discussion to provide support for the effect observed or to hypothesize why an effect was not observed; in 5 of these 11 studies [40,47-50], theory was presented only in a post hoc manner, with the theory being introduced for the first time in the discussion. Only two studies [51,52] used theoretical constructs to develop the evaluation and only one study [46] used theory throughout at least five categories of use (justification of the study, conceptualization and development of the intervention, making predictions, and discussion of results using theory post hoc).

**Table 2:** Category of theory use summary

**Category**	**Count**
Justification	6
Intervention Design	13*
Pilot testing	0
Evaluation	2
Predictions	10
Post hoc	11
Other	0

Note: Categorization in multiple categories is possible.

Justification: Theory is discussed in the background/literature review/objectives section and is used to support study design/purpose. Intervention Design: Theory informed the intervention, either conceptually or by specifically influencing the design of the intervention. Pilot Testing: Theory was utilized to guide pilot testing of the intervention. Evaluation: Theory or constructs outlined in the theory were used to guide outcomes measurement or develop the evaluation strategy. Predictions: At least one stated purpose of the study is to test the influence of a variable predicted to be relevant based on a given theory. Post hoc: Theory is discussed in the discussion section for the purposes of supporting or explaining the results of the study.

*In 1 of the 13 studies that fell into this category, a specific indication of how the theory was used to design the intervention was not offered.

N = 20 studies.

Those studies that used theory ranged in publication year from 1982 to 2010 with no obvious trend towards greater use within any given year or years during that time.

Of the 13 studies (9%)[38,39,42-44,46,49,51-56] that clearly used theory to develop the A&F intervention, 12 offered specific indications of how that was done. The thirteenth study [44] only indicated that the theory contributed to the intervention without explicit mention of the specific ways in which it did so. Similar to the overall use of theory, the two most commonly used theories for intervention development were Bandura’s Social Cognitive Theory (n = 4), and Rogers’ Diffusion of Innovations (n = 3). Of particular interest is that seven of the papers that utilized theory for intervention development discussed the theory in the introduction only, not in the study methods.

While there was range in the degree of detail provided as to how the theory informed the intervention, a few examples show varying approaches to this task. Sommers and colleagues [46] described an Organizational Development Theory that suggests provider participation in setting norms as an approach to increasing behavior change. Their intervention was one of the few that was specifically designed to incorporate and test this theory by having an experimental group that participated in developing their own performance norms. In another study, an Adult Learning Theory that encourages interactive features as an approach to facilitate provider behavior change was used to create an A&F intervention that encouraged the active participation of providers receiving the feedback [54]. de Almeida Neto and colleagues[52] borrowed principles from Bandura’s Social Cognitive Theory to develop an A&F intervention that provided immediate feedback to maximize generalization and refine skills, and positive attainment and corrective information to increase confidence and help maintain commitment to the program.

## Discussion

Our aim was to establish the extent, type and purpose to which theories have been explicitly used in the literature on one of the most widely used interventions in implementation science: A&F. All 140 studies that were included in the most recent Cochrane review on the effectiveness of A&F were reviewed and represent comprehensive, high quality data with which to examine this issue. Our results indicate that theory is rarely reported in this literature. Our systematic review found that 20 (14% of) studies explicitly reported using theory to some degree, and of these, 13 (9%) explicitly used a theory to inform development of the intervention. Our criteria for determining theory use were not overly stringent in that we erred on the side of inclusion, with four studies not meeting all of our criteria for theory use still being included.

By comparison, Davies, Walker and Grimshaw reviewed studies of guideline dissemination and implementation strategies from 1966 to 1998 and found a 6% rate (14 out of 235) of ‘explicit’ theory use [12]. It is difficult to ascertain whether the higher rate of theory use found in our review (6% versus 14%) represents a true difference, differences across intervention type (guidelines versus A&F) or an increase in theory use since 1998. Durand completed a similar investigation on the use of theory for interventions using patient decision aids and also showed relatively poor theory penetrance [57]. This is despite Medical Research Council (MRC) guidelines recommending theory use for complex intervention design [58,59], and an increased prevalence of discourse on the imperative for theory in implementation studies [7,60].

Some authors have investigated theory use in A&F studies by isolating the investigation to one theory, attempting to determine if they could match intervention components to specific theoretical constructs deemed relevant in order to ascertain factors that might contribute to effectiveness. Using Kluger and DeNisi’s Feedback Intervention Theory [61] and Control Theory [62], Hysong [63] and Gardner [6] were able to establish a robust method for their investigations but found that in many cases, interventions were either not theoretically designed or described in enough detail to permit a clear analysis. Of note is that the 2 theories of interest in these theory-based investigations of A&F were not found among the 18 different theories in this review. This is a point made more compelling in that Kluger and DeNisi’s Feedback Intervention Theory [61] is a theory specific to A&F interventions. In addition, of the 20 studies using theory, 18 different theories were employed. Clearly, no consensus exists among empirical researchers on how to approach A&F from a theoretical perspective.

It is not clear that the theories reported as being used in these studies are the most appropriate for designing A&F interventions. While Rogers’ Diffusion of Innovation theory addresses multiple aspects of diffusion, it is relatively silent on the topic of a specific intervention like audit and feedback. Similarly, while Bandura’s Social Cognitive Theory is relevant to many aspects of behavior change and suggests some mechanisms by which feedback works generally, it does not have much specific and direct applicability to audit or to explaining A&F effectiveness in the context of complex health provider behavior change. As a result, it seems that relatively few uses of theory reported in the studies we reviewed were highly suited to the specific intervention being designed and deployed. Theory use was varied across and within each field. Certainly, some relevant theories across the four fields were used but with only single studies using these theories, it is difficult to make useful generalizations about these specific theories for designing A&F interventions.

A range of theory use was found across our nine categories with the greatest use being for intervention development (n = 13), prediction (n = 10), and post hoc explanation (n = 11). While no theories were used to guide field testing, there was also no use of the ‘other’ category, implying that our seven categories were comprehensive. In the event that theory utilization increases, this might not continue to be the case, and more nuanced categorization could be required [64]. Only one study was placed in the intervention design category without offering a specific indication of how theory was used to inform design, implying that if a theory was utilized to develop the intervention, it was typically described with reasonable detail.

A more thorough discussion about the optimal or best practices for employing theory in KT interventions is warranted. Of the 13 studies that used theory to design the intervention, only 5 discussed the study results in the discussion in relation to the theory utilized. This could mean that these were not studies specifically designed to test the theory recommendations. Five studies used theory as a justification for the study design or purpose in the introduction but did not return to discuss the theory past these initial sections, indicating minimal interest in reflecting on study results in relation to the theory. While best approaches to theory use are unknown, utilizing theory to design interventions is likely advantageous [64], and utilizing theory only as a post hoc explanation of observed results is less likely to advance a science measurably. Clearly outlined, consistent and systematic approaches for using theory for intervention design are limited (see Michie [65] and Kok [66] for two exceptions).

As a response to the multitude of theories available to help explain behavior change, and the challenges inherent in choosing specific theories, a broadening of theoretical approaches is occurring in implementation science. Brehaut and Eva have advocated for a ‘menu of constructs’ approach in which the application of theory becomes a process of choosing relevant individual constructs from any number of theories to inform implementation efforts [4]. Michie and colleagues developed a framework that summarized a set of 33 mutually agreed upon theories that were used to inform a core set of 128 constructs critical for behavior change [65]. This core set of constructs was further summarized into 14 key domains relevant to behavior change. Many of these 33 theories are included in the list of theories in this review, but not all. As well, there are theories in our review that were not included in the Michie list.

While it is likely that a wide range of theoretical perspectives will be required to increase our understanding of A&F, at present we cannot make specific recommendations as to which theories to use for designing A&F interventions or on how to use them. Certainly, having more theory-designed interventions that are reported clearly would make synthesis from a theoretical perspective more efficient[3,6] but without a comprehensive approach to understanding the theoretical basis for the effects of A&F, a hit and miss approach to designing A&F interventions will likely prevail [11]. Although a comprehensive list of potential theories and/or causal mechanisms might be impossible to generate, we believe a prioritized list of those believed to be imperative for understanding A&F would be beneficial. The result of such an approach would facilitate a better understanding of priorities for testing different A&F interventions, how to design effective A&F interventions, and what to report in A&F intervention and design.

Several limitations of this study warrant consideration. Our inclusion criteria limited our focus to randomized controlled trials, and we only utilized what was included in the study report alone. It is possible that study authors did incorporate theory into their study, but did not report it in the article, or only provided limited detail. Reviews of studies that have included contacting study authors to augment data collection report value in doing so, but also indicate that the extra effort may or may not have an effect on review outcomes [6]. It is also possible that other study designs may have yielded a different level of detail concerning theory utilization and/or intervention development.

The imperative for theory use in intervention design is supported in principle only [67]; it remains unanswered empirically if using theory for study design or intervention development leads to more rapid and efficient knowledge generation. While too few theory-informed interventions were found in the A&F literature to enable a trustworthy comparison, we support the use of theory in these studies and believe it will provide a means through which to advance implementation science and support synthesis. Certainly, empirical evidence in support of this proposition would be valuable to the field.

## Conclusions

The use of theory in studies of A&F is sparse, particularly for intervention design. No consensus was evident in this review on the types of theories utilized. Advancing our understanding of the theoretical mechanisms in support of A&F and thus optimizing A&F as an intervention will require a closer examination of how theory is utilized in studies of A&F.

## Competing interests

Anne Sales is a co-editor-in-chief and Susan Michie is an associate editor for Implementation Science. Neither of these two authors were involved in the editorial process for this paper and decisions regarding this manuscript were made independently by other Implementation Science editors.

## Authors’ contributions

HLC participated in the design of the study, performed data extraction, participated in analysis, and drafted the manuscript. JCB conceived of the study, and participated in coordination. KC performed data extraction and participated in coordination and analysis. MC performed data extraction. KWE conceived of the study. All authors read, provided feedback, and approved the final manuscript.

## Supplementary Material

Additional file 1: Table S1 The use of theories in studies of audit and feedback: Description and stage of theory use. N=20, Red: Theory used for intervention conception or design. Click here for file
