# Supplementary material for: A systematic review of the use of theory in randomized controlled trials of audit and feedback
Source: Implement Sci. 2013 Jun 10;8:66. doi: 10.1186/1748-5908-8-66 (PMC3702512; doi:10.1186/1748-5908-8-66)
Supplement: Additional file 1: Table S1 — The use of theories in studies of audit and feedback: Description and stage of theory use. N=20, Red: Theory used for intervention conception or design. [file 1748-5908-8-66-S1.docx]

# Additional File 1

Additional File 1 - The use of theories in studies of audit and feedback: Description and stage of theory use

N=20

Red: Theory used for intervention conception or design. [an alternative is to italicize]

| No | Paper (first author, year) | Theory | Theory type | Reference | Location in paper | Category of Theory Use |
| --- | --- | --- | --- | --- | --- | --- |
| 1 | Grady, 1997 [17] | Social Cognitive Theory  Referred to as “Theoretical Framework of Behavioral Psychology” | Psychology | Bandura, A. Principles of Behaviour Modification. New York: Holt Rinehart & Winston, 1969. | Introduction | Justification of the work  Guiding conception of the intervention  Development of the intervention  Predictions |
| 2 | Sommers,  1984[25] | OrganizationDevelopment Theory | Organization | Levitt HJ. Applied organizational change in industry: structural, technological and humanistic approaches. In: March JH, ed. Handbook of organizations. Chicago: Rand, 1965;1152. | Introduction  Discussion | Justification of the work  Guiding conception of the intervention  Development of the intervention  Predictions  Post hoc discussion |
| 3 | Bonevsky1999[32] | Diffusion of Innovations | Diffusion | Rogers, E. Diffusion of innovations. New York: Free Press, 1983 | Methods Discussion | Guiding conception of the intervention  Development of the intervention  Post hoc discussion |
| 4 | Bahranai, 2004[20] | Social Cognitive Models | Psychology | Bonetti D, et al., Can psychological models bridge the gap between clinical guidelines and clinicians' behaviour: a randomised controlled trial of an intervention to influence intention to implement evidence-based practice. Br Dent J 2003; 195: 403-407.  Put as social cognitive models: undefined. | Methods Discussion | Predictions  Post hoc discussion |
| 5 | Foy, 2004  [18] | Theory of Planned Behaviour | Psychology | Reference for theory not cited (used TPB). | Methods Discussion | Guiding conception of the intervention  Development of the intervention  Post hoc discussion |
| 6 | Hayes, 2002[28] | 1. Technology Diffusion Theory  2. Organizational Readiness model  3. Readiness for Change Model | 1.Diffusion  2. Org  3.Psychology | 1. Penland, T. A model to create 'organizational readiness' for the successful implementation of total quality management in hospitals. Int J Qual Health Care. 1997;9:69-72.  2. Rogers, E. Diffusion of Innovations. 4th Ed, New York:The Free Press; 1995.  3. Prochaska JO, DiClemente CC. Transtheoretical therapy: Toward a more integrative model of change. Psychother Theory Res Pract. 1982;20:161-173. | Discussion | Post hoc discussion |
| 7 | Tierney, 1986[29] | Theory of Reasoned Action | Psychology | 1. Fishbein, A Ajzen, I. Belief, attitude, intention, and behaviour: An introduction to theory and research. Reading, MA: Addison-Wesley, 1968. | Discussion | Post hoc discussion |
| 8 | Mold, 2008[22] | Diffusion of Innovations | Diffusion | Rogers, E. The Change agent. In: Rogers, E. (ed). Diffusion of Innovations, 4th ed. New York (NY): Simon and Schuster, The Free Press; 1995:335-70. | Introduction | Justification of the work  Guiding conception of the intervention  Development of the intervention  Predictions  Note: Rogers was used to support the use of practice facilitation in the intervention but not the A&F component of the intervention. |
| 9 | Phillips, 2005[33] | Adult Learning Theory | Learning | Knowles, M. Andragogy in Action: Applying modern principles of adult learning: San Francisco, Jossey-Bass, 1984. | Methods | Guiding conception of the intervention  Development of the intervention |
| 10 | Goff, 2003[34] | 1a/b. Theory of Reasoned Action  Social Cognitive Theory  2. Diffusion Theory | 1a/b. Psychology  2. Diffusion | 1a. Fishbein,M. A theory of reasoned action: some applications and implications. Nebr Symp Motiv 1979;27:65-116.  1b. Bandura, A. Social foundation of thought and action: a social-cognitive theory. Englewood Cliffs, NJ: Prentice-Hall: 1986.  2. Rogers, EM. Diffusion of innovations. New York: Free Press: 1983. | Methods | Guiding conception of the intervention  Development of the intervention |
| 11 | Sinclair, 1982[23] | Lawler's organizational theory | Org | Lawler, EE. Control systems in organizations: In Dunette MD (ed): Handbook of Industrial and Organizational Psychology. Chicago: rand McNally, 1976. pp 1247-1291. | Introduction  Discussion | Justification of the work  Guiding conception of the intervention  Predictions  Post hoc discussion |
| 12 | Cheater, 2006[26] | Trans-theoretical Model | Psychology | Prochaska JO, DiClemente CC, Norcross JC. In search of how people change: applications to addictive behaviours. Am Psychologist. 1992;47: 1102–1114. | Discussion | Post hoc discussion |
| 13 | de Almeida Neto, 2000[31] | 1. Social Cognitive Theory  2. Stages of Change Model | 1. 2. Psychology | 1. Bandura, A. Social Foundations of Thought and Action: A social cognitive theory. Englewood Cliffs, NJ: Prentice-Hall; 1986.  2. Prochaska JO, DiClemente CC. Towards a comprehensive model of change. In Miller WR, Heather N, eds. Treating addictive behaviours. New York: Plenum, 1986:3-27. | Introduction | Guiding conception of the intervention  Development of the intervention  Evaluation  Predictions  Note: Bandura was used for the health provider component of the intervention and Prochaska & DiClemente was used for the patient component of the intervention. |
| 14 | Siriwardena 2002 [24] | Adult learning theory | Learning | Knowles, MS. Adult learner: a neglected species. Houston, TX: Gulf Publishing, 1990. | Introduction | Justification of the work |
| 15 | Lagerlov, 2000[21] | 1. Behavioral Psychology  2. Self-efficacy  3. Theory of Medical education | 1.2. Psychology  3. Learning | 1. Ullman & Krasner. A psychological approach to abnormal behaviour. Gap 12: Behaviour Modification. New Jersey: Prentice-Hall, 1975: 224-47.  2. Bandura, A. Self-efficacy. The exercise of control. New York: WH Freeman, 1997:3.  3. Coles, C & Holm, HA. Learning in medicine: Towards a theory of medical education. Oslo: Scandinavian University Press, 1993: 189-209. | Introduction | Justification of the work  Guiding conception of the intervention  Development of the intervention  Predictions |
| 16 | Avery, 2010[30] | 1. Human Error Theory  2a/b. Diffusion of Innovation | 1. Psychology  2a/b. Diffusion and Org | 1. Reason J. Human error: models and management. British Medical Journal.  2000 Mar;320:768-70.  2a. Rogers E. Diffusion of innovations. New York: Free Press1983.  2b. Greenhalgh T, Robert G, Macfarlane F, Bate P, Kyriakidou O. Diffusion of  Innovations in Service Organizations: Systematic Review and  Recommendations. The Milbank Quarterly. 2004;82:581. | Introduction  Methods | Guiding conception of the intervention  Development of the intervention  Evaluation (the two diffusion theories only)  Predictions  Note: Human error was used to design the intervention. Diffusion theory was used as a framework for the qualitative analysis of interviews asking participants for their impression of the intervention. |
| 17 | Scholes, 2006[35] | Precede/  Proceed planning model | Psychology | Green, L.W., & Kreuter, M.W. *Health Promotion Planning.*Mayfield Publishing Company. CA:Mayfield;1991. | Methods | Guiding conception of the intervention  Development of the intervention  Predictions |
| 18 | Hayes, 2001[28] | Social Learning Theory | Learning | Perry, C., Baranowski, T., Parcel, GS. How individuals, environments, and health behaviour interact: Social Learning Theory. In Health Behaviour and Health Education: Theory, Research, and Practice. San Francisco: Jossey-Bass Publishers, 1990. | Methods  Discussion | Guiding conception of the intervention  Development of the intervention  Predictions  Post hoc discussion |
| 19 | Foster, 2007[27] | Kirkpatrick's Hierarchy of Levels of Evaluation | Other (Evaluation) | Kirkpatrick, DI. Evaluation of training. In Training and development handbook Edited by: Craig, R, Birrel, I. New York: McGraw-Hill: 1967. | Discussion | Post hoc discussion |
| 20 | Kiefe, 2001[19] | social cognitive models | Psychology | Conner, M, & Norman, P. The role of cognition in health behaviours. In: Conner M, Norman P, eds. Predicting Health Behaviour. Buckingham, England: Open University Press; 1996. | Discussion | Post hoc discussion |

Notes: Introduction (includes introduction, background, literature review, objectives), Discussion (includes conclusion).
